# 5-[(4-Acetyl­phenyl)­aminomethyl­ene]-2,2-dimethyl-1,3-dioxane-4,6-dione

**DOI:** 10.1107/S1600536809017425

**Published:** 2009-05-14

**Authors:** Rui Li, Zhen-Yu Ding, Yu-Quan Wei, Jian Ding

**Affiliations:** a State Key Laboratory of Biotherapy, West China Hospital, Sichuan University, Chengdu 610041, People’s Republic of China; b State Key Laboratory of Drug Research, Shanghai Institute of Materia Medica, Chinese Academy of Sciences, Shanghai, 201203, People’s Republic of China

## Abstract

In the title compound, C_15_H_15_NO_5_, the six-membered dioxane ring assumes an envelope conformation with the dimethyl substituted C atom as the flap atom. An intra­molecular N—H⋯O inter­action is also present. In the crystal structure the mol­ecules are linked *via* C—H⋯O hydrogen bonds into supra­molecular chains along the *b* axis.

## Related literature

For the biological activity of 4(1*H*)-quinolone structures, see: Ruchelman *et al.* (2003 ▶). 5-Aryl­amino­methyl­ene-2,2-dimethyl-1,3-dioxane-4,6-diones are key inter­mediates in the synthesis of 4(1*H*)quinolone derivatives by thermolysis (Cassis *et al.*, 1985 ▶).
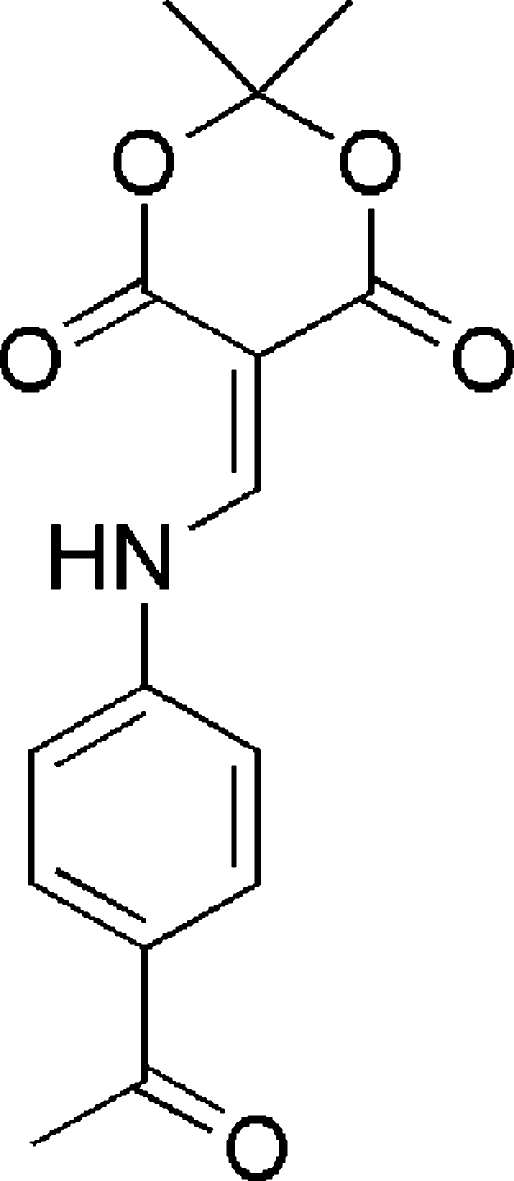


## Experimental

### 

#### Crystal data

C_15_H_15_NO_5_
*M* *_r_* = 289.28
Triclinic, 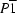

*a* = 7.102 (3) Å
*b* = 7.356 (4) Å
*c* = 13.856 (4) Å
α = 82.79 (4)°
β = 83.19 (4)°
γ = 86.03 (4)°
*V* = 712.0 (5) Å^3^
*Z* = 2
Mo *K*α radiation
μ = 0.10 mm^−1^
*T* = 292 K
0.44 × 0.36 × 0.32 mm

#### Data collection

Enraf–Nonius CAD-4 diffractometer
Absorption correction: none
2894 measured reflections
2621 independent reflections
1399 reflections with *I* > 2σ(*I*)
*R*_int_ = 0.008
3 standard reflections every 100 reflections intensity decay: 1.8%

#### Refinement

*R*[*F* ^2^ > 2σ(*F* ^2^)] = 0.064
*wR*(*F* ^2^) = 0.179
*S* = 1.03
2621 reflections
197 parameters
H atoms treated by a mixture of independent and constrained refinement
Δρ_max_ = 0.23 e Å^−3^
Δρ_min_ = −0.34 e Å^−3^

### 

Data collection: *DIFRAC* (Gabe & White, 1993 ▶); cell refinement: *DIFRAC*; data reduction: *NRCVAX* (Gabe *et al.*, 1989 ▶); program(s) used to solve structure: *SHELXS97* (Sheldrick, 2008 ▶); program(s) used to refine structure: *SHELXL97* (Sheldrick, 2008 ▶); molecular graphics: *ORTEP-3* (Farrugia, 1997 ▶); software used to prepare material for publication: *SHELXL97*.

## Supplementary Material

Crystal structure: contains datablocks I, New_Global_Publ_Block. DOI: 10.1107/S1600536809017425/xu2521sup1.cif

Structure factors: contains datablocks I. DOI: 10.1107/S1600536809017425/xu2521Isup2.hkl

Additional supplementary materials:  crystallographic information; 3D view; checkCIF report

## Figures and Tables

**Table 1:** Hydrogen-bond geometry (Å, °)

*D*—H⋯*A*	*D*—H	H⋯*A*	*D*⋯*A*	*D*—H⋯*A*
N1—H1*N*⋯O4	0.84 (4)	2.05 (3)	2.699 (3)	133 (2)
C1—H1*B*⋯O3^i^	0.96	2.57	3.480 (4)	158
C9—H9⋯O5^i^	0.93	2.59	3.429 (4)	150

Symmetry code: (i) 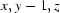.

**Fig. 1.**
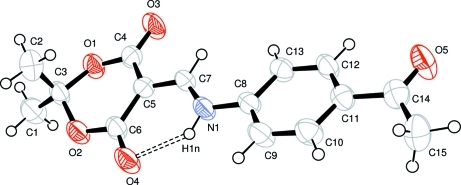
The molecular structure of the title compound, with displacement ellipsoids drawn at the 30% probability level.

## supplementary crystallographic information

### Comment

The 4(1*H*)quinolone structure plays an extremely important role in the field of pharmaceutical chemistry. These compounds have been used as precursors for anticancer agents, anti-malarial agents and reversible (H+/K+) ATPase inhibitors (Ruchelman *et al.*, 2003). 5-Arylaminomethylene-2,2-dimethyl-1,3-dioxane-4,6-diones are the key intermediates which can be used to synthesize the 4(1*H*)quinolone derivatives by thermolysis (Cassis *et al.*, 1985).

The molecular structure is shown in Fig. 1. The six membered dioxane ring assumes an envelope conformation. The imino group links with the adjacent O atom *via* O—H···O hydrogen bonding. In the crystal structure the molecules are linked *via* C—H···O hydrogen bonding into one dimensional supra-molecualr chain along the *b* axis (Table 1).

### Experimental

A methanol solution (50 ml) of Meldrum's acid (1.44 g, 0.01 mol) and methylorthoformate (1.27 g, 0.012 mol) was heated to reflux for 2 h, then the arylamine (1.35 g, 0.01 mol) was added into the above solution. The mixture was heated under reflux for another 8 h and then filtered. Single crystals were obtained from the filtrate after 2 d.

### Refinement

The imino H atom was located in a difference Fourier map and refined isotropically. Other H atoms were positioned geometrically with C—H = 0.93 (aromatic) or 0.96 Å (methyl), and refined using a riding model with *U*_iso_(H) = 1.5*U*_eq_(C) for methyl and 1.2*U*_eq_(C) for the others.

### Crystal data

**Table:**

C_15_H_15_NO_5_	*Z* = 2
*M**_r_* = 289.28	*F*(000) = 304
Triclinic, *P*1	*D*_x_ = 1.349 Mg m^−^^3^
Hall symbol: -P 1	Mo *K*α radiation, λ = 0.71073 Å
*a* = 7.102 (3) Å	Cell parameters from 25 reflections
*b* = 7.356 (4) Å	θ = 4.4–7.4°
*c* = 13.856 (4) Å	µ = 0.10 mm^−^^1^
α = 82.79 (4)°	*T* = 292 K
β = 83.19 (4)°	Block, colourless
γ = 86.03 (4)°	0.44 × 0.36 × 0.32 mm
*V* = 712.0 (5) Å^3^	

### Data collection

**Table:**

Enraf–Nonius CAD-4 diffractometer	*R*_int_ = 0.008
Radiation source: fine-focus sealed tube	θ_max_ = 25.5°, θ_min_ = 1.5°
graphite	*h* = −8→8
ω/2θ scans	*k* = −3→8
2894 measured reflections	*l* = −16→16
2621 independent reflections	3 standard reflections every 100 reflections
1399 reflections with *I* > 2σ(*I*)	intensity decay: 1.8%

### Refinement

**Table:**

Refinement on *F*^2^	Primary atom site location: structure-invariant direct methods
Least-squares matrix: full	Secondary atom site location: difference Fourier map
*R*[*F*^2^ > 2σ(*F*^2^)] = 0.064	Hydrogen site location: inferred from neighbouring sites
*wR*(*F*^2^) = 0.179	H atoms treated by a mixture of independent and constrained refinement
*S* = 1.03	*w* = 1/[σ^2^(*F*_o_^2^) + (0.0987*P*)^2^] where *P* = (*F*_o_^2^ + 2*F*_c_^2^)/3
2621 reflections	(Δ/σ)_max_ < 0.001
197 parameters	Δρ_max_ = 0.23 e Å^−^^3^
0 restraints	Δρ_min_ = −0.34 e Å^−^^3^

### Special details

**Table:**

Geometry. All e.s.d.'s (except the e.s.d. in the dihedral angle between two l.s. planes) are estimated using the full covariance matrix. The cell e.s.d.'s are taken into account individually in the estimation of e.s.d.'s in distances, angles and torsion angles; correlations between e.s.d.'s in cell parameters are only used when they are defined by crystal symmetry. An approximate (isotropic) treatment of cell e.s.d.'s is used for estimating e.s.d.'s involving l.s. planes.
Refinement. Refinement of *F*^2^ against ALL reflections. The weighted *R*-factor *wR* and goodness of fit *S* are based on *F*^2^, conventional *R*-factors *R* are based on *F*, with *F* set to zero for negative *F*^2^. The threshold expression of *F*^2^ > σ(*F*^2^) is used only for calculating *R*-factors(gt) *etc*. and is not relevant to the choice of reflections for refinement. *R*-factors based on *F*^2^ are statistically about twice as large as those based on *F*, and *R*- factors based on ALL data will be even larger.

### Fractional atomic coordinates and isotropic or equivalent isotropic displacement parameters (Å^2^)

**Table:**

	*x*	*y*	*z*	*U*_iso_*/*U*_eq_	
O1	0.4606 (3)	0.5224 (2)	0.87501 (13)	0.0548 (6)	
O2	0.4847 (3)	0.3153 (3)	0.75537 (13)	0.0585 (6)	
O3	0.5363 (3)	0.8104 (3)	0.83800 (15)	0.0713 (7)	
O4	0.5696 (3)	0.4044 (3)	0.60005 (14)	0.0688 (7)	
O5	0.9300 (3)	1.4570 (3)	0.25900 (17)	0.0767 (7)	
N1	0.6950 (3)	0.7445 (3)	0.54722 (17)	0.0485 (6)	
H1N	0.674 (4)	0.644 (5)	0.528 (2)	0.066 (9)*	
C1	0.3511 (5)	0.2250 (4)	0.9154 (2)	0.0677 (9)	
H1A	0.3605	0.2286	0.9836	0.101*	
H1B	0.3674	0.1003	0.9010	0.101*	
H1C	0.2284	0.2754	0.8999	0.101*	
C2	0.6988 (5)	0.2750 (5)	0.8795 (2)	0.0708 (9)	
H2A	0.7894	0.3472	0.8371	0.106*	
H2B	0.7236	0.1478	0.8706	0.106*	
H2C	0.7094	0.2910	0.9463	0.106*	
C3	0.5022 (4)	0.3356 (4)	0.8554 (2)	0.0504 (7)	
C4	0.5331 (4)	0.6609 (4)	0.8097 (2)	0.0503 (7)	
C5	0.5861 (4)	0.6195 (3)	0.71100 (18)	0.0440 (6)	
C6	0.5512 (4)	0.4421 (4)	0.6835 (2)	0.0486 (7)	
C7	0.6522 (4)	0.7568 (3)	0.64119 (19)	0.0443 (7)	
H7	0.6681	0.8692	0.6625	0.053*	
C8	0.7584 (4)	0.8835 (4)	0.47359 (19)	0.0443 (7)	
C9	0.8222 (4)	0.8338 (4)	0.3821 (2)	0.0561 (8)	
H9	0.8262	0.7113	0.3711	0.067*	
C10	0.8804 (4)	0.9674 (4)	0.3063 (2)	0.0562 (8)	
H10	0.9233	0.9337	0.2447	0.067*	
C11	0.8752 (4)	1.1500 (4)	0.3217 (2)	0.0475 (7)	
C12	0.8132 (4)	1.1971 (4)	0.4143 (2)	0.0510 (7)	
H12	0.8113	1.3192	0.4256	0.061*	
C13	0.7541 (4)	1.0661 (4)	0.4903 (2)	0.0491 (7)	
H13	0.7118	1.0998	0.5521	0.059*	
C14	0.9294 (4)	1.2990 (4)	0.2415 (2)	0.0558 (8)	
C15	0.9783 (5)	1.2522 (5)	0.1391 (2)	0.0731 (10)	
H15A	1.0042	1.3622	0.0958	0.110*	
H15B	0.8735	1.1954	0.1195	0.110*	
H15C	1.0885	1.1690	0.1365	0.110*	

### Atomic displacement parameters (Å^2^)

**Table:**

	*U*^11^	*U*^22^	*U*^33^	*U*^12^	*U*^13^	*U*^23^
O1	0.0800 (14)	0.0361 (11)	0.0476 (11)	0.0014 (9)	−0.0018 (10)	−0.0097 (9)
O2	0.0936 (15)	0.0361 (11)	0.0467 (11)	−0.0137 (10)	−0.0013 (10)	−0.0087 (9)
O3	0.125 (2)	0.0320 (11)	0.0598 (12)	−0.0023 (11)	−0.0121 (12)	−0.0163 (9)
O4	0.1213 (19)	0.0381 (11)	0.0475 (12)	−0.0173 (12)	0.0040 (11)	−0.0130 (9)
O5	0.0993 (18)	0.0370 (13)	0.0893 (17)	−0.0106 (11)	0.0019 (13)	0.0012 (11)
N1	0.0641 (16)	0.0299 (13)	0.0525 (15)	−0.0091 (11)	−0.0043 (12)	−0.0071 (11)
C1	0.087 (2)	0.056 (2)	0.0591 (19)	−0.0135 (17)	0.0026 (17)	−0.0095 (16)
C2	0.078 (2)	0.053 (2)	0.078 (2)	0.0051 (16)	−0.0068 (18)	0.0021 (16)
C3	0.071 (2)	0.0296 (14)	0.0505 (16)	0.0001 (13)	−0.0032 (14)	−0.0068 (12)
C4	0.0647 (19)	0.0389 (16)	0.0488 (16)	0.0057 (13)	−0.0135 (14)	−0.0092 (13)
C5	0.0512 (16)	0.0371 (15)	0.0455 (15)	0.0007 (12)	−0.0106 (13)	−0.0086 (12)
C6	0.0670 (19)	0.0346 (15)	0.0435 (16)	−0.0027 (13)	−0.0018 (13)	−0.0065 (12)
C7	0.0527 (16)	0.0300 (14)	0.0525 (17)	−0.0017 (12)	−0.0121 (13)	−0.0086 (12)
C8	0.0449 (16)	0.0374 (15)	0.0520 (16)	−0.0071 (12)	−0.0067 (12)	−0.0067 (12)
C9	0.069 (2)	0.0416 (16)	0.0597 (18)	−0.0133 (14)	0.0005 (15)	−0.0137 (14)
C10	0.0639 (19)	0.0526 (19)	0.0525 (17)	−0.0094 (15)	0.0036 (14)	−0.0140 (14)
C11	0.0434 (16)	0.0431 (16)	0.0548 (17)	−0.0042 (12)	−0.0029 (13)	−0.0029 (13)
C12	0.0608 (19)	0.0312 (14)	0.0611 (18)	−0.0040 (12)	−0.0069 (14)	−0.0048 (13)
C13	0.0588 (18)	0.0377 (15)	0.0518 (16)	0.0021 (13)	−0.0048 (13)	−0.0129 (13)
C14	0.0464 (17)	0.0531 (19)	0.066 (2)	0.0003 (14)	−0.0062 (14)	−0.0001 (15)
C15	0.083 (2)	0.063 (2)	0.065 (2)	−0.0021 (18)	0.0077 (18)	0.0072 (16)

### Geometric parameters (Å, °)

**Table:**

O1—C4	1.361 (3)	C5—C7	1.374 (4)
O1—C3	1.438 (3)	C5—C6	1.450 (4)
O2—C6	1.342 (3)	C7—H7	0.9300
O2—C3	1.434 (3)	C8—C9	1.380 (4)
O3—C4	1.216 (3)	C8—C13	1.389 (4)
O4—C6	1.212 (3)	C9—C10	1.390 (4)
O5—C14	1.217 (3)	C9—H9	0.9300
N1—C7	1.315 (3)	C10—C11	1.383 (4)
N1—C8	1.408 (3)	C10—H10	0.9300
N1—H1N	0.85 (3)	C11—C12	1.384 (4)
C1—C3	1.498 (4)	C11—C14	1.495 (4)
C1—H1A	0.9600	C12—C13	1.383 (4)
C1—H1B	0.9600	C12—H12	0.9300
C1—H1C	0.9600	C13—H13	0.9300
C2—C3	1.499 (4)	C14—C15	1.497 (4)
C2—H2A	0.9600	C15—H15A	0.9600
C2—H2B	0.9600	C15—H15B	0.9600
C2—H2C	0.9600	C15—H15C	0.9600
C4—C5	1.438 (3)		
			
C4—O1—C3	119.3 (2)	O2—C6—C5	117.1 (2)
C6—O2—C3	120.1 (2)	N1—C7—C5	126.2 (2)
C7—N1—C8	127.6 (2)	N1—C7—H7	116.9
C7—N1—H1N	116 (2)	C5—C7—H7	116.9
C8—N1—H1N	116 (2)	C9—C8—C13	120.2 (3)
C3—C1—H1A	109.5	C9—C8—N1	117.8 (3)
C3—C1—H1B	109.5	C13—C8—N1	122.0 (2)
H1A—C1—H1B	109.5	C8—C9—C10	119.7 (3)
C3—C1—H1C	109.5	C8—C9—H9	120.1
H1A—C1—H1C	109.5	C10—C9—H9	120.1
H1B—C1—H1C	109.5	C11—C10—C9	120.7 (3)
C3—C2—H2A	109.5	C11—C10—H10	119.7
C3—C2—H2B	109.5	C9—C10—H10	119.7
H2A—C2—H2B	109.5	C10—C11—C12	118.8 (3)
C3—C2—H2C	109.5	C10—C11—C14	122.5 (3)
H2A—C2—H2C	109.5	C12—C11—C14	118.7 (3)
H2B—C2—H2C	109.5	C13—C12—C11	121.3 (3)
O2—C3—O1	111.4 (2)	C13—C12—H12	119.4
O2—C3—C1	105.9 (2)	C11—C12—H12	119.4
O1—C3—C1	106.4 (2)	C12—C13—C8	119.3 (3)
O2—C3—C2	110.2 (2)	C12—C13—H13	120.4
O1—C3—C2	109.4 (2)	C8—C13—H13	120.4
C1—C3—C2	113.4 (3)	O5—C14—C11	120.4 (3)
O3—C4—O1	117.5 (2)	O5—C14—C15	120.4 (3)
O3—C4—C5	125.9 (3)	C11—C14—C15	119.2 (3)
O1—C4—C5	116.5 (2)	C14—C15—H15A	109.5
C7—C5—C4	118.8 (2)	C14—C15—H15B	109.5
C7—C5—C6	120.4 (2)	H15A—C15—H15B	109.5
C4—C5—C6	120.5 (2)	C14—C15—H15C	109.5
O4—C6—O2	118.4 (2)	H15A—C15—H15C	109.5
O4—C6—C5	124.5 (3)	H15B—C15—H15C	109.5

### Hydrogen-bond geometry (Å, °)

**Table:**

*D*—H···*A*	*D*—H	H···*A*	*D*···*A*	*D*—H···*A*
N1—H1N···O4	0.84 (4)	2.05 (3)	2.699 (3)	133 (2)
C1—H1B···O3^i^	0.96	2.57	3.480 (4)	158
C9—H9···O5^i^	0.93	2.59	3.429 (4)	150

Symmetry codes: (i) *x*, *y*−1, *z*.
